# The frequency of adverse drug reaction related admissions according to method of detection, admission urgency and medical department specialty

**DOI:** 10.1186/1472-6904-9-8

**Published:** 2009-05-04

**Authors:** Miran Brvar, Nina Fokter, Matjaz Bunc, Martin Mozina

**Affiliations:** 1 Poison Control Centre, University Medical Centre Ljubljana, Ljubljana, Slovenia; 2 Medical Faculty, University of Ljubljana, Ljubljana, Slovenia; 3 Department for Cardiology, University Medical Centre Ljubljana, Ljubljana, Slovenia; 4 Slovenian National Pharmacovigilance Centre, University Medical Centre Ljubljana, Ljubljana, Slovenia

## Abstract

**Background:**

Adverse Drug Reactions (ADRs) have been regarded as a major public health problem since they represent a sizable percentage of admissions. Unfortunately, there is a wide variation of ADR related admissions among different studies. The aim of this study was to evaluate the frequency of ADR related admissions and its dependency on reporting and method of detection, urgency of admissions and included medical departments reflecting department/hospital type within one study.

**Methods:**

The study team of internal medicine specialists retrospectively reviewed 520 randomly selected medical records (3%) of patients treated in the medical departments of the primary city and tertiary referral governmental hospital for certain ADRs causing admissions regarding WHO causality criteria. All medical records were checked for whether the treating physicians recognised and documented ADRs causing admissions. The hospital information system was checked to ensure ADR related diagnoses were properly coded and the database of a national spontaneous reporting system was searched for patients with ADRs included in this study.

**Results:**

The established frequency of admissions due to certain ADRs recognised by the study team and documented in medical records by the treating physicians was the same and represented 5.8% of all patients (30/520). The frequency of ADR causing admissions detected by employing a computer-assisted approach using an ICD-10 coding system was 0.2% (1/520), and no patient admitted due to ADRs was reported to the national reporting system (0/520). The recognized frequency of ADR related admissions also depends on the department's specialty (p = 0.001) and acceptance of urgently admitted patients (p = 0.001). Patients admitted due to ADRs were significantly older compared to patients without ADRs (p = 0.025). Gastrointestinal bleeding due to NSAID, acetylsalicylic acid and warfarin was the most common ADR that resulted in admission and represented 40% of all certain ADRs (12/30) according to WHO causality criteria.

**Conclusion:**

ADRs cause 5.8% of admissions in medical departments in the primary city and tertiary referral hospital. The physicians recognise certain ADR related admissions according to WHO causality criteria and note them in medical records, but they rarely code and report ADRs. The established frequency of ADR related admissions depends on the detection method, department specialty and frequency of urgently admitted patients.

## Background

Adverse drug reactions (ADRs) have been regarded as a major public health problem since they represent a sizable percentage of admissions and an economic burden [1,2]. Unfortunately, there is a wide variation of ADR related admissions among different European studies. In a UK study of ADR related admissions in all hospitals using the computer-assisted International Classification of Diseases (ICD) coding system ADR related admissions represented 0.5% of total hospital admissions [3], while in a prospective observation study in only two hospitals with medical and surgical departments ADR related admissions corresponded to 5.2% of total hospital admissions [4]. In a similar prospective observation, European studies performed in medical departments admissions due to certain ADRs according to WHO (World Health Organisation) criteria encounter 3.2% in France [5] and 6.2% in Germany [6] of all admitted patients, while in a prospective computerised "event monitoring" study in internal medicine departments in Swiss general and teaching hospitals admissions due to ADRs encounter 3.3% [7].

Studies in some even more specialised departments give the highest percentages of patients with ADRs on admission, for example, up to 27.4% of patients had at least one possible, likely or very likely ADR on admission in medical intensive care units in France [8]. After comparing different studies we can assume that a wide variation in the frequency of ADR related admissions could be the result of different detection methods and specialties of the included departments and hospitals [9].

The aim of this study was to evaluate the frequency of ADR related admissions and its dependency on the method of reporting and detection, urgency of admissions and included medical departments within one study.

## Methods

### Patients

The study was conducted in medical departments for adult patients of the governmental University Medical Centre Ljubljana (UMCL), the primary city hospital for the Slovenian capital city of Ljubljana serving a population of 400,000 inhabitants and tertiary referral hospital centre serving a population of 2,000,000. It includes nine specialised medical departments with 82 regular beds dedicated to gastroenterology, 74 beds cardiology, 50 beds angiology, 42 beds endocrinology, 32 beds nephrology, 26 beds haematology, 14 beds intensive care, 13 beds pulmonology, and 6 beds to toxicology. Patients with solid tumours are treated mainly at the Institute of Oncology and were not included in the study. The patients are admitted to medical departments through a medical emergency department that covers all medical departments, through outpatient departments of individual specialised medical departments and those transferred from other primary hospitals and non-medical departments of UMCL, or whose admittance was planned for special diagnostic procedures or therapy.

### Date collection

In the study a team of three internal medicine specialists, of whom one was a postgraduate in clinical pharmacology, retrospectively reviewed 520 complete medical records with all patient medical documents chosen at random by a computer for ADRs causing admissions. The number of included patients was computed according to the main aim of the study, which was the evaluation of ADR related admissions depending on the method of reporting and detection (3 possible answers), urgency of admissions (4 possible answers) and medical departments (9 possible answers). 16 possible answers regarding the method of reporting and detection, path of admission and medical department were multiplied by 30 cases per each answer, giving us 480 patients. Finally, 480 patients were rounded up to 520, which represents 3% of 17,500 patients hospitalised in medical departments in UMCL in 2006.

The definition of ADRs used in the study was that of the WHO: "Any noxious and unintended response to a drug that occurs at doses normally used in humans for the prophylaxis, diagnosis or therapy of disease" [10]. The assessment of ADR causality was performed also using the WHO system [11]. The study team had to reach a consensus decision on the certainty of ADR related admission and excluded doubtful cases. ADR causing admissions were considered as certain if ADR had a plausible time relationship to drug intake, plausible response to withdrawal, definitive pharmacological or phenomenological explanation and could not be explained by disease or other drugs [11]. Patients with ADR on admission who were not directly responsible as well as those who received a deliberate or unintentional overdose or relapsed because of non-compliance were not considered to have ADR related admission. Afterwards all certain cases of ADR related admissions regarding WHO causality criteria were classified according to ADR type. The type of ADR estimated its preventability and suggested preventive measures for each ADR such as closer patient clinical and laboratory monitoring, applying protective measures and other selection of drugs. These were checked for their feasibility in every patient individually by the study team revising patient medical documentation.

For all patients age, sex, number of prescribed drugs on admission, discharge diagnosis with their ICD-10 codes performed by treating physicians, alcohol abuse, renal failure, liver failure, urgency and path of admittance and subspecialty of medical department were recorded. Renal failure was considered in patients with serum creatinine levels 50% above the upper normal level (150 micromol/L) and liver failure in patients with liver enzymes (alanine aminotransferase, aspart aminotransferase and gamma glutamyltransferase) at twice the upper normal level.

ADR causing admissions, documented and described in the medical records by the treating physicians who were unaware of the study and not specially trained and encouraged to record, code or report ADRs, were carefully recorded and additionally reassessed by the study team, who met to reach a consensus decision on the certainty of ADR related admission according to WHO causality criteria documented by the treating physicians and excluded doubtful cases.

Subsequently the hospital information system based on ICD-10 coding was checked for whether ADR related diagnoses causing admission of included patients were properly coded. ICD-10 codes including the words "drug induced" or "due to" a medicine or which are recognised to be invariably caused by a drug along with external cause codes indicating that a drug was implicated were considered as an ADR related diagnosis. The results of ADR coding are expressed as the percentage of ADR related admissions documented by the treating physician that were not properly coded and the percentage of ADR related admissions in patients included in this study that would be established only by searching a hospital information system based on ICD-10 coding.

Finally, the Slovenian national spontaneous ADR reporting system database was searched for patients with ADR reported by UMCL in 2006. The Slovenian National Pharmacovigilance Centre was established in 1984 and has been a member of Eudravigilance since 2004. The treating physicians should report all ADRs to the national reporting system using the ADR reporting form, but reporting is spontaneous and no repercussion is followed for not reporting. In 2006 the National Pharmacovigilance Centre received 100 ADR reports per one million inhabitants, mainly from hospital physicians. 20 ADR reports were sent to the National Pharmacovigilance Centre from UMCL in 2006. The patients in the national database of reported ADRs and patients included in this study were matched according to date, reporting hospital, patient's initials, age, drugs and reported ADRs. If all matched well, we assumed this to be the same case. The results of reporting ADR related admissions to the national reporting system are expressed as a percentage of ADRs described by the treating physician in medical records that were not reported to the national spontaneous reporting system and a percentage of ADR related admissions in patients included in this study that would be established only by searching the national spontaneous reporting system database.

A Slovenian National Medical Ethics Committee approved this study.

### Statistical analysis

The data is presented as the mean and range unless otherwise indicated. Pearson's correlation was used to correlate age, number of drugs and number of diagnoses. The analysis of variance and Bonferroni correction method were used to analyse the differences in admissions due to ADRs between established urgency and paths of admission and subspecialty of included departments. Statistics of patient characteristics between patients admitted due to ADR and patients without ADR on admission were compiled by multivariate logistic regression analysis. A p value of less than 0.05 was considered to be significant.

Analyses were carried out with the Statistical Package for Social Science 11.5 for Windows (SPSS).

## Results

### Study population characteristic

Over one year, from 1 January to 31 December 2006, 17,230 patients were admitted to the medical departments of UMCL. The study included 520 patients, which represented 3% of all admitted patients. The overall study of population characteristics is given in Table 1. Age correlated with number of drugs (p = 0.001) and number of diagnoses (p = 0.001).

**Table 1:** Characteristics of the study population (N = 520)

Male *	296 (57%)
Age **	65.6 (19–94)

Number of drugs on admission **	4.4 (0–16)

Number of discharge diagnoses **	5.0 (1–14)

Renal failure *	50 (9.6%)

Liver failure *	52 (10.0%)

Alcohol abuse *	36 (6.9%)

* Number of patients and percentage; **mean and range

### Admissions due to ADRs detected in patient medical records by a study team

The prevalence of admissions due to certain ADRs according to WHO causality criteria detected in medical records by the study team of internal medicine specialists was 5.8% (30 of 520 admitted patients).

The difference in admissions due to ADRs between the established urgency and paths of admission was significant (p = 0.001) *in global*, but the *post hoc *test results showed that only ADR related urgent admissions through the medical emergency department were significantly different from planned admittance (p = 0.001), with a much higher percentage of admissions (Table 2).

**Table 2:** Urgency and paths of admission to medical departments

	**No. of patients admitted regardless of ADR (N = 520)**	**No. of patients admitted due to ADR (%) (N = 30)**
Urgent admissions through Medical Emergency Department	210	23 (11.0%) *

Urgent admissions through Medical Outpatients Department	48	3 (6.3%)

Urgent transfer from other primary hospitals or departments	49	4 (8.2%)

Non-urgent/planned admissions	213	0 (0%) *

* p = 0.001; urgent admission through Medical Emergency Department v. non-urgent/planned admissions

The difference in admissions due to ADRs between the established departments was significant (p = 0.001) *in global *as well, but the *post hoc *test results showed that only ADR related admissions to the department of gastroenterology were significantly different from the departments of cardiology (p = 0.001) and angiology (p = 0.001), with a much higher percentage of admissions (Table 3).

**Table 3:** Departments of admittance of the study population

**Medical departments**	**No. of patients admitted regardless of ADR (N = 520)**	**No. of patients admitted due to ADR (%) (N = 30)**
Cardiology	163	4 (2.5%) *

Angiology	74	0 (0%) **

Gastroenterology	72	13 (18.0%) *, **

Endocrinology	68	6 (8.8%)

Haematology ***	47	4 (8.5%)

Intensive Care Medicine	38	2 (5.3%)

Nephrology	32	1 (3.1%)

Pulmonology	21	0 (0%)

Toxicology	5	0 (0%)

* p = 0.001 Department of Gastroenterology v. Department of Cardiology, ** p = 0.001 Department of Gastroenterology v. Department of Angiology, *** haematology includes only patients with leukaemia and some lymphomas

The characteristics of patients according to ADR on admission are presented in Table 4. Patients admitted due to ADRs were significantly older compared to patients without ADRs (p = 0.025) (Table 4).

**Table 4:** Characteristics of patients according to ADR on admission

	**No-ADR**	**A-ADR**	**p**
Number	490 (94.3%)	30 (5.7%)	

Men	281 (57.3%)	15 (50.0%)	0.494

Age *	65.2 (19–94)	71.5 (42–89)	0.025

Number of drugs *	4.3 (0–16)	5.6 (1–12)	0.273

Number of diagnoses *	5.0 (1–14)	5.5 (1–10)	0.674

Renal failure	46 (9.4%)	4 (13.3%)	0.419

Liver failure	50 (10.2%)	2 (6.7%)	0.859

Alcohol abuse	34 (6.9%)	2 (6.7%)	0.195

Death	19 (3.9%)	0 (0%)	0.998

No-ADR patients without ADR; A-ADR patients admitted due to ADR; * mean and range.

Types of ADRs and drug classes that resulted in admission are presented in Table 5. All ADRs are already known and labelled. Gastrointestinal bleeding due to NSAID, acetylsalicylic acid and warfarin was the most common ADR that resulted in admission and represented 40% of all ADRs (12/30). Cardiovascular medications were involved in 20% of ADRs (6/30), antineoplastic and immunosuppressive agents in 13% (4/30), corticosteroids in 13% (4/30) and antidiabetic drugs in 10% of ADRs (3/30).

**Table 5:** Description of certain ADRs as a cause of admittance

**ADR (No. of patients)**	**Class of drugs (No. of patients with ADR/No. of patients with potentially preventable ADR)**
Cardiovascular disorders	
Bradycardia (4)	Beta-adrenergic blocker (2/2) *
	Beta-adrenergic blocker and digoxin (2/2) *

Gastrointestinal disorder	
Gastrointestinal bleeding (12)	Acetylsalicylic acid (4/4) *
	Acetylsalicylic acid with NSAID (1/1) *
	NSAID (5/4) *
	Warfarin (2/2) *
Liver failure (1)	Antifungal agent (1/0)

Renal disorders	
Renal failure (1)	Antineoplastic agent (1/0)

Haematological disorders	
Anaemia (2)	Antineoplastic agent (1/1) *
	Immunosuppressive agent (1/1) *
Pancytopenia (1)	Antineoplastic agent (1/1) *

Metabolic disorders	
Hypoglycaemia (3)	Insulin (2/2) *
	Insulin and sulphonyurea (1/1) *
Hyperglycaemia (4)	Corticosteroid (4/4) *
Hypokaliemia (1)	Diuretic (1/1) *
Hyperkaliemia (1)	ACE inhibitor and spironolactone (1/1) *

* Drug dose related ADR (type A)

### Admissions due to ADRs documented in patient medical records by treating physicians

The frequency of admissions due to certain ADRs according to WHO causality criteria recognised and documented in medical records by the treating physicians was 5.8% of all patients (30/520), the same as detected in medical records by the study team (Table 5). The study team did not find any incorrectly described or unrecognised certain ADR according to WHO causality criteria causing admissions by the treating physicians.

### Admissions due to ADRs detected in the hospital information system by a computer-assisted approach using ICD-10 coding

Among 30 patients admitted due to certain ADRs according to WHO causality criteria in this study only one patient (3.3%) had an ADR related disorder properly coded by ICD-10 (K710 Drug induced liver disease) in the hospital computer assisted information system. The under-coding rate of ADRs in this study was 96.7% (29/30) and prevalence of admissions due to ADR detected by the computer-assisted approach using the ICD-10 coding system only 0.2% (1/520).

### Admissions due to ADRs reported to the national ADR spontaneous reporting system

No patient admitted due to certain ADRs according to WHO causality criteria in this study was reported to the national ADR reporting system. The under-reporting rate in this study was 100% (30/30) and prevalence of admissions due to ADRs reported to the national ADR spontaneous reporting system was 0% (0/520).

## Discussion

Admissions to medical departments due to certain ADRs according to WHO causality criteria recognised and documented in medical records by treating physicians and confirmed by the study team in this primary city and tertiary referral hospital with about 60% of urgently admitted patients was found in 5.8% of patients, which is similar to studies performed in other European primary city and tertiary referral hospitals [6].

In this study the established percentage of ADR related admissions primarily depends on the method used to detect patients admitted due to ADRs since treating physicians recognised and documented in medical records all certain ADRs according to WHO causality criteria, but they rarely coded ADR related diagnoses according to ICD-10 for the hospital information system and never reported them to the authorities. Accordingly the frequency of ADR related admissions found in the hospital computer assisted information system based on ICD-10 codes and the database of the spontaneous reporting system might grossly underestimate the burden of drug-induced disorders as a cause of hospital admission as was also shown in other studies of information systems using computer assisted coding [3,12,13] and spontaneous reporting systems [13-16], where a spontaneous underreporting system of up to 100% was established as in this study [17]. The limitation of this study is the low number of patients admitted due to ADR, which prevents us from determining spontaneous reporting with a frequency below 3.3% that could still be useful for signal detection, particularly for unlabelled ADRs.

In 2006 a spontaneous reporting system in UMCL was clearly not effective and various initiatives have been introduced to encourage and facilitate reporting of ADRs such as grated accessibility to the ADR database through electronic reporting, education at both undergraduate and postgraduate level, and rewarding reporters who supply good quality ADR reports with credit points for continuing education as well as feedback information, whose effectiveness in increasing reporting have been suggested and proved in other studies [16,18]. On the other hand, one of the main reasons for hospital computer assisted information system underestimation of ADR burden as a cause of hospital admission is limitations of the coding system for identifying ADRs [13,19,20]. In particular, the ICD-10 codes which clearly define a "drug-induced" diagnosis are clearly not comprehensive in their scope [13].

Additionally ADR related diagnoses and particularly their ICD-10 codes may, also be inaccurate, and since physicians might consider they are used only for administrative purposes, they may be less concerned with accurate recording of ICD codes [21].

Future integration of computer systems within hospitals and the expansion of electronic prescribing and electronic health records may make ICD codes a more useful practical tool [19]. Hospitals should also consider interventions such as developing educational interventions and mechanisms to provide feedback on recording of ADR to hospital physicians and to improve the identifications and coding of admissions linked to ADR [3]. However, in future it would be necessary to develop coding and reporting systems independently of treating physicians that are usually not specially trained to code diagnoses and who feel great resistance to coding and reporting overwhelming and well-known type A reactions [14,22]. This could be achieved by employing specially trained medical experts that understand the coding system and reporting to the authorities. Instead of using ICD-10 codes, they could code ADRs according to The Medical Dictionary for Regulatory Activities (MedDRA), which is already used in the National Pharmacovigilance Centre, because a key requirement is a more detailed breakdown of drugs implicated in ADRs [3] and the inclusion of causality assessment criteria by which the likelihood of ADR being related to a particular drug can be estimated [3].

Additionally, the established frequencies of ADR related admissions depend on the subspecialties of medical departments and acceptance of urgently admitted patients with the highest prevalence of ADR related admissions to the Gastroenterology Department through the Medical Emergency Department. The highest prevalence of ADR related admissions to the Gastroenterology Department was the consequence of gastrointestinal bleeding due to NSAID, which is the fifth most commonly used drug class in Slovenia according to defined daily dose [23]. However, with regard to these results, we should bear in mind that antineoplastic drugs against solid tumours, which are also a common cause of ADR, were not included in this study. Therefore studies of ADR related admissions might give very different results depending on the frequency of urgently admitted patients through the emergency department and subspecialty of included departments reflecting the type of hospital (primary v. tertiary) and the acceptance of different ADR related disorders.

In this study older patients were more likely to develop ADRs causing hospital admission, as has already been presented in some similar studies [4,17,24,25], and should also be considered comparing different studies because those including younger patients might present a lower incidence of ADR related admissions [26]. 5.8% of patients admitted due to ADRs in medical departments raise the obligatory question of their prevention. 80% or even more admissions due to ADRs are supposed to be potentially avoidable because they are dose related reactions, and thus predictable from the known pharmacology of the drug by closer patient clinical and laboratory monitoring, applying protective measures, selecting other drugs and patient education [1,14,27-29]. Accordingly, in this study 90% (27/30) of ADRs were potentially preventable. However, the cost benefit and feasibility of such preventive procedures is questionable since no decrease in the percentage of admittances due to ADR was found during the last decade, despite knowledge of their possible prevention. The reason could be that potentially preventable dose related ADRs often happen at low drug doses where further dose reduction is not possible. One of the frequently mentioned potentially preventable dose related ADRs is gastrointestinal bleeding due to acetylsalicylic acid, but acetylsalicylic acid is mainly used in small antitrombotic doses and further dose reduction is not reasonable. Nevertheless, physicians and managers must be aware of the reality of ADRs and focus further preventive effort on certain drug classes that cause serious or common potentially preventable drug related admissions such as NSAID, acetylsalicylic acid, beta-adrenergic blocker, diuretic, angiotensin-converting enzyme inhibitor, sulphonyurea, digoxin, corticosteroide and insulin [24] that also correspond to 82% (28/34) of drug classes causing ADR related admissions in this study. Although the preventability is disputable, this study can help us to identify the most frequent ADRs related to hospitalisations and target these ADRs to take preventive action [14].

## Conclusion

ADRs cause 5.8% of admissions in medical departments in the primary city and tertiary referral governmental hospital. The physicians recognise and document in medical records all certain ADRs causing admission according to WHO causality criteria, but they rarely code ADRs by ICD-10 codes in a hospital computer assisted information system and spontaneously report them to the authorities. The established frequency of ADR related admissions depends, besides on the detection method, also on the specialty of departments and frequency of urgently admitted patients reflecting hospital type.

## Competing interests

The authors declare that they have no competing interests.

## Authors' contributions

MB, MM and MB analysed medical records. MB and NF extracted and analysed the data and drafted the manuscript. MB and MM participated in the design of the study and co-ordination and helped to draft the manuscript. All authors contributed to the final version of the manuscript, provided full access to the data presented, and had final responsibility for the decision to submit it for publication.

## Pre-publication history

The pre-publication history for this paper can be accessed here:


